# Intrauterine Transfusion Complicated by Umbilical Artery Thrombosis

**DOI:** 10.1155/2019/5952326

**Published:** 2019-02-20

**Authors:** Roopali V. Donepudi, Kenneth J. Moise

**Affiliations:** Department of Obstetrics, Gynecology and Reproductive Medicine, Division of Maternal-Fetal Medicine, UT Health-School of Medicine at Houston, The Fetal Center, Children's Memorial Hermann Hospital, Houston, TX, USA

## Abstract

**Background:**

Fetal anemia results from several conditions; however intrauterine transfusion (IUT) remains the treatment for severe cases. The complications of this procedure are rare and yet can result in preterm delivery or fetal death.

**Case:**

31 y/o G3P2002 with Rh alloimmunization underwent IUT from 19 to 35 weeks. Umbilical artery thrombosis was noted after her 5^th^ IUT. Further transfusions were performed without any complications and she delivered a full term male infant with APGARS of 8 and 9 at 1 and 5 minutes, respectively.

**Conclusion:**

The complication of umbilical artery thrombosis is unusual and the optimal management is unclear. We report such a case and propose that the presence of Hyrtl's anastomosis near the placental cord insertion may explain the reassuring fetal status throughout the pregnancy.

## 1. Introduction

The incidence of fetal anemia secondary to Rhesus alloimmunization has decreased since the implementation of Rh immunoglobulin prophylaxis in Rh-negative women. However, intrauterine transfusion (IUT) continues to be the standard treatment for fetal anemia. Typically this is undertaken by accessing the umbilical vein near the placental cord insertion. The procedure is not without complications including preterm premature rupture of membranes (PPROM), preterm labor (PTL), preterm birth (PTB), infection, and cord complications including cord hematoma, bleeding, and fetal bradycardia [1]. We report a case of umbilical artery thrombosis after intrauterine transfusion with delivery at term without any further complications. We obtained consent for publication by the patient.

## 2. Case Presentation

The patient was a 31 y/o Gravida 3 Para 2 who presented to our center at 19-week gestation. She had two prior uncomplicated full term vaginal deliveries and received Rh immunoglobulin during and after each of her previous pregnancies. She has no significant past medical or surgical history.

During this pregnancy, her first trimester studies revealed an anti-D titer of 2048. The fetal status was noted to be RHD positive on amniocentesis.

On her initial evaluation at 19-week gestation at our center, the middle cerebral artery (MCA) Doppler revealed a peak systolic velocity (PSV) of 2.37 MoM. There was mild ascites and cardiomegaly.

After counseling the patient underwent the first in a series of combined intravascular/intraperitoneal intrauterine transfusions (see Table 1). The ascites and cardiomegaly resolved after the second transfusion.

**Figure 1:**
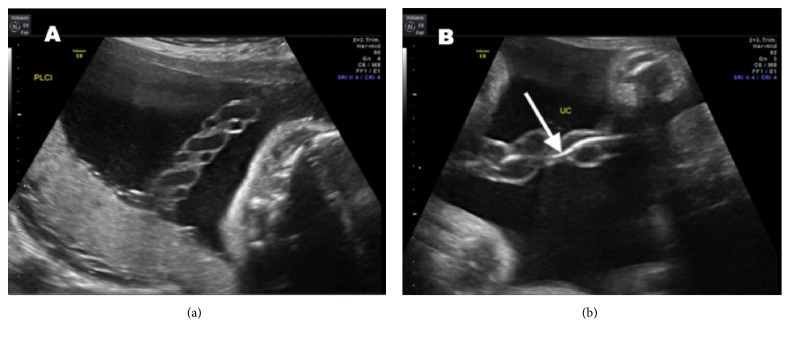
(a) Three-vessel cord with two patent umbilical arteries was noted on initial evaluation. (b) Thrombosed umbilical artery (arrow) is seen in this segment of the cord.

The fourth transfusion was complicated by an episode of transient bradycardia with spontaneous recovery after removal of the procedure needle from the umbilical vein. On a preoperative ultrasound prior to her sixth procedure, thrombosis of one of the umbilical arteries was noted (see Figure 1). A review of earlier ultrasounds indicated two patent umbilical arteries although visualization of the cord was not specifically undertaken postoperatively after the fourth procedure or before and after the fifth procedure. Based on the reassuring status of the fetus, a decision was made to continue serial intrauterine transfusions. Antenatal testing was initiated with weekly biophysical profiles and daily kick counts.

In addition to fetal anemia, this pregnancy was complicated by diet controlled gestational diabetes and mild polyhydramnios with an AFI of 29. The estimated fetal weight at 35 weeks ultrasound was 3193gms (87^th^  %ile). She underwent a cesarean section at 37 weeks, delivering a 3480-gram male fetus in vertex presentation with APGARS of 8 and 9 at 1 and 5 minutes, respectively. After delivery the umbilical cord was examined and a 3-vessel cord with an intraluminal hematoma in one umbilical artery was confirmed. The hematocrit was 37% after birth. The total bilirubin was 9.9mg/dL and the direct bilirubin was 2.6 mg/dL. As per our neonatal protocols weekly hematocrit levels were monitored which remained above 30%; hence no transfusions were needed during this period.

## 3. Discussion

In pregnancies complicated by Rh alloimmunization for the first time fetal status should be confirmed by cell free DNA or fetal RHD gene testing on amniocentesis in cases of paternal heterozygosity or unknown status. These cases are managed conservatively initially by following serial antibody titers that should ideally be done in the same laboratory since variations in titers among laboratories commonly occurs. Once critical titers are exceeded MCA Dopplers should be performed to identify fetuses that are anemic. Serial titers are no longer monitored after reaching this critical titer. When the MCA PSV is > 1.5 MoM [2] for gestational age, a fetal blood sampling and intrauterine transfusion is done when anemia is confirmed. This procedure is performed between 18 and 35 weeks, as cases <18 weeks are limited by the small size of the vessels, thereby increasing the complications. We perform a combination of intravascular, in the umbilical vein, and intraperitoneal transfusions as this provides a more stable hematocrit and increases the duration between transfusions thereby reducing total number of procedures [3]. The technical aspects of performing an IUT have been previously described by the coauthor (KJM) [4]. Serial MCA Dopplers have been reported to be unreliable following an IUT especially after 2 transfusions. Therefore, we do not use these Dopplers to determine the timing of subsequent transfusions. The second, third, and subsequent IUTs are performed at 7-, 14-, and 21-day intervals, respectively, based on the expected drop in the hemoglobin level each day [4, 5]. The procedure-related loss after IUT has been reported to be 2% [6]; however there is limited information on the incidence of umbilical artery thrombosis and further management if this occurs.

We report a case of severe fetal anemia secondary to Rh alloimmunization requiring multiple intrauterine transfusions starting at 19 weeks. The bradycardia noted during the fourth transfusion may have been secondary to umbilical arterial puncture. We suspect that the thrombosis of the umbilical artery may be secondary to this inadvertent puncture while attempting to insert the needle in to the umbilical vein for the IUT. Although her treatment course was complicated by thrombosis of one of the umbilical arteries, subsequent transfusions were successful resulting in a term delivery.

Smith et al. [7] described a similar case. After two intravascular transfusions, a single umbilical artery was seen on ultrasound. They attempted another transfusion 4 weeks later, which was complicated by fetal bradycardia with the need for discontinuation of the procedure. Several weeks later, during an intravascular transfusion, the fetus developed refractory bradycardia and the infant was delivered by emergent cesarean section. The infant weighed 1920gms and had APGAR scores of 4, 2, and 6 at 1, 5, and 10 minutes, respectively. On pathology, coagulative necrosis and luminal thrombosis of one of the umbilical arteries were noted.

We believe that the presence of Hyrtl anastomosis explains the favorable outcome in our case. Joseph Hyrtl first described this anastomosis in 1870 as a communication between the umbilical arteries near the placental surface. This anastomosis is seen in 95% of all placentas [8]. It is believed to play a role in equalizing the blood pressure between the different parts of the placenta supplied by the umbilical arteries [9] and was thought to be protective in cases of compression or occlusion of one artery. This is particularly important late in pregnancy during uterine contractions when compression of one umbilical artery is well tolerated.

Gordon et al. [10] performed a quantitative analysis of the hemodynamic characteristics of the flow through this anastomosis. They demonstrated the importance of the presence of Hyrtl's anastomosis for the regulation of blood flow in discordant arteries. The anastomosis resulted in a redistribution of flow from the unaffected artery to the affected artery and equilibration of pressure gradients in the arteries to improve placental perfusion.

One of the most common umbilical cord anomalies is the presence of a single umbilical artery (SUA) that can be detected early by ultrasound. In cases of isolated SUA, fetal growth restriction and increased perinatal mortality has been reported [11]. Suess et al. [12] found an increase in the size of this isolated umbilical artery with gestational age, which may be an adaptive mechanism to enhance the placental perfusion. Hyrtl anastomosis is absent in these cases of SUA. The absence of the Hyrtl anastomoses near the placental cord insertion and failure of the arterial dilatation are likely responsible for the poor placental perfusion and complications like growth restriction and perinatal mortality. When the umbilical cord has two umbilical arteries, initially the loss of one vessel can be compensated for by the Hyrtl anastomosis. Adequate perfusion of the placenta may still be possible in these cases.

In conclusion, we suggest that while the detection of thrombosis of one of the umbilical arteries following IUT deserves a cautious approach, continuing the pregnancy and future transfusions may be a reasonable option especially in the case of prematurity and reassuring fetal status.

## Figures and Tables

**Table 1:** Intraoperative fetal transfusion data.

Gestational Age (weeks)	Initial Hct (%)	Final Hct (%)	IVT volume (mL)	IPT volume (mL)	Complication
19.1	6	24	8	5	None

19.6	22.4	37.6	10	10	None

21.6	30.5	40.9	12	20	None

25	27.8	30.1	15	Postponed	Fetal bradycardia

25.1				50	None

28.1	29.2	45.7	50	80	None

31.6	28.6	Unable to obtain final Hct due to dislodged needle.	70	115	2VC noted pre-op.

35	26	40.7	100	150	None
